# Comparative Assessment of Large Language Model Outputs and NHS Patient Information in Oral Medicine

**DOI:** 10.7759/cureus.90242

**Published:** 2025-08-16

**Authors:** Jamila Tukur Jido, Ahmed Al-Wizni, Mariam Alkateb, Noor Salem

**Affiliations:** 1 Department of Geriatrics, Queen Elizabeth University Hospital, Glasgow, GBR; 2 Department of Internal Medicine, Broomfield Hospital, Essex, GBR; 3 Department of Dentistry, King's College London, London, GBR; 4 Department of Dentistry, Walmsley Dental, Manchester, GBR

**Keywords:** artificial intelligence, health literacy, large language models, national health service, nhs guidelines, patient education, readability measures

## Abstract

Background

Artificial intelligence (AI) and large language models (LLMs) offer transformative potential in healthcare communication, with the National Health Service (NHS) Long Term Plan envisioning digital tools to support accessible, patient-centred information. However, whether LLM-generated health materials are sufficiently readable for patient use remains uncertain, particularly in oral medicine, where conditions like xerostomia, oral candidiasis, and sialolithiasis are common.

Objective

This study compared the readability of patient information leaflets generated by three LLMs with the NHS UK patient leaflets on common oral medicine conditions, to assess their suitability for public health communication.

Methods

A cross-sectional analysis was conducted, in which each LLM was prompted to produce patient leaflets for xerostomia, oral candidiasis, and sialolithiasis. Outputs were compared to the NHS UK leaflets on identical topics. Texts were analysed using established readability metrics: Flesch Reading Ease Score (FRES), Flesch-Kincaid Grade Level (FKGL), and Gunning Fog Index. Results were summarised descriptively without formal statistical testing due to the exploratory study design.

Results

The NHS UK leaflets consistently demonstrated superior readability across all conditions and metrics, with lower FKGL scores (5.9-6.3) and higher FRES scores (70.5-72.4), indicating suitability for readers aged 11-14 years (Key Stage 3). Among LLMs, ChatGPT produced the most readable outputs, with FKGL scores ranging from 6.8 to 7.2. DeepSeek outputs were moderately more complex (FKGL: 8.3-8.7), while Gemini generated the most complex texts (FKGL: 9.7-10.2), often exceeding recommended reading levels for patient materials.

Conclusion

While LLMs, especially ChatGPT, show promise in generating patient information, their outputs remain less readable than professionally authored NHS materials. Given that nearly half of UK adults may struggle with complex health texts, the higher reading levels required for LLM-generated content could impede patient understanding and exacerbate health inequalities. As AI becomes more integrated into healthcare communication, ensuring that AI-generated materials meet established readability standards is essential to support equitable, patient-centred care.

## Introduction

Advancements in artificial intelligence (AI) and large language models (LLMs) are set to transform healthcare delivery [1]. The latest National Health Service (NHS) Long Term Plan sets out goals for the next decade, in which AI is to be embedded across health services to improve efficiency, personalise care, and empower patients [2]. Central to this vision is the commitment to providing patients with clear and accessible health information that supports self-management and shared decision-making. This priority reflects a growing recognition that digital health tools must not only produce clinically accurate content but also communicate in a manner comprehensible to people with diverse levels of general and health literacy [3-5].

LLMs such as ChatGPT (OpenAI, San Francisco, CA), DeepSeek (Hangzhou, China), and Gemini (Google, Mountain View, CA) are tools capable of generating health-related content. There is considerable interest in harnessing these technologies to supplement patient education, enhance clinical communication, and address information gaps [6,7]. However, it remains unclear how the language produced by these AI systems compares with current patient information resources.

This issue is especially relevant for common oral medicine conditions such as xerostomia, oral candidiasis, and sialolithiasis. Given the prevalence of these conditions across medical, dental, and pharmacy settings, patient-facing health information must be not only scientifically accurate but also written in accessible language [8-10]. Readability is a key determinant of effective health communication [11], referring to how easily a reader can process and understand written material. Several validated readability metrics have been developed to assess this attribute. The Flesch Reading Ease Score (FRES) and the Flesch-Kincaid Grade Level (FKGL) estimate the education level required for comprehension by analysing sentence length and word complexity [12,13]. The Gunning Fog Index similarly quantifies the linguistic demands of written materials, with higher scores indicating greater reading difficulty [14,15].

Despite growing interest in AI-generated health content, there is currently a significant gap in the literature regarding the readability of LLM outputs, particularly in relation to oral medicine [16,17]. To address this gap, our research group has undertaken an analysis of the readability of AI-generated content from ChatGPT, DeepSeek, and Gemini. By applying established readability indices, our study aims to evaluate whether the information produced by these models is suitable for patient use and consistent with the NHS Long Term Plan’s objective of delivering digital health solutions that are inclusive and equitable.

## Materials and methods

Study design and data generation

This study was conducted as a cross-sectional comparative analysis to evaluate the readability of patient information materials generated by ChatGPT (GPT-4), DeepSeek (V3), and Gemini (Flash 2.5). These outputs were compared to publicly available patient information leaflets from NHS UK [18-20]. This study focuses on three common oral medicine conditions: xerostomia, oral candidiasis, and sialolithiasis. These models were selected because they were, at the time of the study, widely available, in active public use, and capable of generating detailed patient information without requiring paid institutional licences. Their inclusion allowed for comparison across distinct AI platforms while maintaining a feasible scope for this exploratory analysis.

Each LLM was prompted with the identical standardised query: “Please write me a patient information leaflet on (insert condition)”. Before this prompt, the search history and cache for each LLM were erased. Each model produced one output for each condition, generating a total of nine texts for analysis. For comparison, patient information leaflets corresponding to these same conditions were sourced from the NHS UK website. NHS UK pages were accessed via a Google search conducted in June 2025. NHS UK materials were selected as the reference standard because they are publicly accessible, professionally authored, and intended for patient use, making them a suitable benchmark for evaluating the potential public usability of AI-generated health information.

All LLM outputs and NHS UK leaflets were copied verbatim into plain text files to preserve the original wording for subsequent analysis.

Pre-processing of text data

Before performing readability analysis, all text outputs underwent standardisation to minimise the influence of non-linguistic formatting features on readability calculations. These pre-processing steps were performed manually to ensure uniformity and accuracy across all documents. No changes were made to the substantive wording of the texts or sentence structure. Due to the exploratory nature of this study and the finite number of outputs obtainable from each LLM in a single session, no formal sample size calculation was conducted.

Readability metrics

To assess the readability of patient information materials, several established readability formulas were employed.

The Flesch Reading Ease Score (FRES) evaluates textual complexity based on sentence length and the number of syllables per word. Scores range from 0 to 100, with higher scores indicating easier reading material [21].

The Flesch-Kincaid Grade Level (FKGL) is closely related to FRES but expresses readability as a US school grade level. FKGL calculates the approximate educational grade required to comprehend the text, based on average sentence length and average syllables per word [22].

The Gunning Fog Index estimates the years of formal education needed to understand a passage on first reading. It factors in average sentence length and the percentage of complex words, defined as words with three or more syllables. A Fog Index of 12 indicates that the material is suitable for a high school senior [23].

These metrics were chosen because they collectively capture different aspects of textual complexity, including sentence structure, word length, and the prevalence of technical or polysyllabic vocabulary, which are especially relevant for patient-facing health information.

All readability scores were calculated using Python (version 3.11; Python Software Foundation, Wilmington, Delaware) and the textstat library (version 0.7.3), which applies standard algorithms for each metric.

Statistical analysis

This study was conducted as an observational, descriptive analysis. For each oral health condition (xerostomia, oral candidiasis, and sialolithiasis), readability scores were calculated for patient information leaflets generated by ChatGPT (GPT-4), DeepSeek (V3), and Gemini (Flash 2.5), and compared descriptively to those from NHS UK materials.

No formal statistical hypothesis testing was performed due to the small sample size (n = 3 per group) and the descriptive, exploratory nature of the study. Instead, differences in readability metrics between LLM outputs and NHS UK materials were summarised using tabular presentation and narrative description.

The analysis aimed to provide initial insights into readability patterns and identify potential gaps in the suitability of AI-generated patient information for public use, rather than to make inferential claims or generalisations.

Ethical considerations

This study did not involve human participants, patient records, or animal subjects. All data analysed were non-identifiable textual outputs generated by publicly accessible AI systems or publicly available NHS UK patient information leaflets. As such, ethical approval was not required. No sensitive or personal information was collected or processed.

Data availability

The textual data analysed in this study consist of non-identifiable outputs generated by publicly accessible LLMs and publicly available patient information leaflets from the NHS UK, accessible via references [18-20]. All documents were produced or accessed during May and June 2025 using identical query prompts or search methods. The compiled texts from LLMs were saved as Word documents (Microsoft Corporation, Redmond, WA) and are available from the corresponding author upon reasonable request.

## Results

Xerostomia

Table 1 shows the comparative readability scores across three metrics for each LLM and the NHS UK leaflet on xerostomia. Readability analysis demonstrated notable differences between the outputs of LLMs and the NHS UK leaflet. Among the LLMs, ChatGPT generated the most readable text overall, with an FRES of 66.9 and an FKGL score of 7, indicating a reading level suitable for individuals aged approximately 11-12 years (UK Year 7). DeepSeek produced text of intermediate complexity, with an FRES score of 58.7 and an FKGL of 8.3, while Gemini generated the most complex material, reflected in a lower FRES of 51.1 and a higher FKGL of 9.7. The Gunning Fog Index confirmed these patterns, with ChatGPT scoring 8.2, DeepSeek 9.9, and Gemini 11.2, suggesting progressively higher reading levels required for comprehension.

**Table 1:** Comparative readability metrics of large language model (LLM) outputs and the NHS UK patient leaflet on xerostomia. Readability scores for health-related text generated by ChatGPT, DeepSeek, and Gemini compared with the NHS UK leaflet on xerostomia. FRES: Flesch Reading Ease Score; FKGL: Flesch-Kincaid Grade Level.

LLM/source	FRES	FKGL	Gunning Fog
ChatGPT	66.9	7	8.2
DeepSeek	58.7	8.3	9.9
Gemini	51.1	9.7	11.2
NHS UK	71.8	6.3	8.1

In comparison, the NHS UK leaflet on dry mouth demonstrated the highest readability overall. It achieved an FRES score of 71.8, indicating text that is fairly easy to read, and an FKGL of 6.3, placing it below the recommended maximum reading level for patient materials (Key Stage 3). Its Gunning Fog Index was 8.1, further supporting its relative accessibility.

Collectively, these results indicate that while ChatGPT produced the most readable content among the LLMs, the NHS UK leaflet remained superior across most readability measures, suggesting greater suitability for public use, particularly among readers with lower literacy levels.

Oral candidiasis

Readability analysis for patient information leaflets on oral candidiasis revealed notable differences between outputs generated by LLMs and the NHS UK leaflet. Among the LLMs, ChatGPT produced the most readable text, achieving an FRES of 67.5 and an FKGL of 6.8, suggesting suitability for readers around 11-12 years of age. DeepSeek’s output was moderately more complex, with an FRES score of 58.0 and an FKGL of 8.5, indicating a higher reading level. Gemini generated the most complex material, reflected in a lower FRES of 50.2 and a substantially higher FKGL of 9.9.

In comparison, the NHS UK leaflet on oral candidiasis was the most readable overall. It achieved the highest FRES of 72.4, indicating material that is relatively easy to read, and the lowest FKGL of 5.9, consistent with a reading level below Key Stage 3.

These findings indicate that while ChatGPT produced the most readable text among the LLMs for oral candidiasis, the NHS UK leaflet consistently exhibited greater readability across all metrics, suggesting it remains more appropriate for public use. Table 2 presents these comparative readability scores across all indices.

**Table 2:** Comparative readability metrics of large language model (LLM) outputs and the NHS UK patient leaflet on oral candidiasis. Readability scores for health-related text generated by ChatGPT, DeepSeek, and Gemini compared with the NHS UK patient leaflet on oral candidiasis, assessed using the Flesch Reading Ease Score (FRES), Flesch-Kincaid Grade Level (FKGL), and Gunning Fog Index.

LLM/source	FRES	FKGL	Gunning Fog
ChatGPT	67.5	6.8	8
DeepSeek	58	8.5	9.7
Gemini	50.2	9.9	11.1
NHS UK	72.4	5.9	8

Sialolithiasis

Readability analysis for patient information leaflets on sialolithiasis (salivary gland stones) demonstrated consistent variation between outputs generated by LLMs and the NHS UK leaflet.

Among the LLMs, ChatGPT produced the most accessible text, with an FRES of 65.8 and an FKGL of 7.2, corresponding to a reading level suitable for individuals around Year 7 in the UK. DeepSeek’s output was moderately more complex, achieving an FRES of 56.7 and an FKGL of 8.7. Gemini generated the most complex text, reflected in a lower FRES of 48.9 and a substantially higher FKGL of 10.2.

In comparison, the NHS UK leaflet on salivary gland stones was the most readable overall. It achieved the highest FRES of 70.5 and the lowest FKGL of 6.1, placing it comfortably below the recommended maximum reading level for patient information materials.

Overall, these results suggest that while ChatGPT produced the most readable text among the LLMs for sialolithiasis, the NHS UK leaflet consistently demonstrated superior readability across all metrics, underscoring its appropriateness for public health communication, particularly for individuals with lower literacy levels. Table 3 provides a detailed comparison of the readability scores for all sources.

**Table 3:** Comparative readability metrics of large language model (LLM) outputs and the NHS UK patient leaflet on sialolithiasis. Readability scores for health-related text generated by ChatGPT, DeepSeek, and Gemini compared with the NHS UK patient leaflet on sialolithiasis, assessed using the Flesch Reading Ease Score (FRES), Flesch-Kincaid Grade Level (FKGL), and Gunning Fog Index.

LLM/source	FRES	FKGL	Gunning Fog
ChatGPT	65.8	7.2	8.4
DeepSeek	56.7	8.7	9.8
Gemini	48.9	10.2	11.5
NHS UK	70.5	6.1	8.3

Visualisation of readability metrics

Readability scores were visualised across all conditions and models to facilitate comparison of LLM outputs with NHS UK patient information leaflets. Bar charts for each readability metric demonstrated consistent patterns across the three clinical conditions: xerostomia, oral candidiasis, and sialolithiasis.

**Figure 1:**
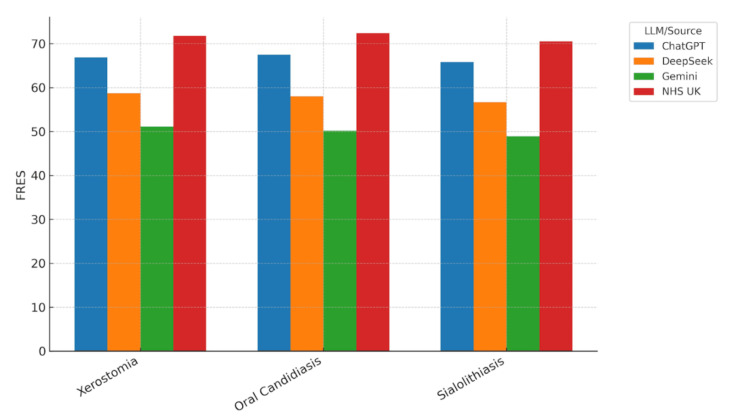
Flesch Reading Ease Score (FRES) for texts on xerostomia, oral candidiasis, and sialolithiasis generated by large language models (LLMs) and the NHS UK patient leaflets. Bar chart comparing Flesch Reading Ease Scores (FRES) across three oral medicine conditions—xerostomia, oral candidiasis, and sialolithiasis—for texts generated by ChatGPT, DeepSeek and Gemini, as well as the NHS UK patient information leaflets. Higher FRES scores indicate easier readability. Across all conditions, NHS UK leaflets consistently demonstrated the highest readability, while Gemini’s outputs showed lower scores, suggesting greater text complexity.

Figure 1 compares the FRES metric. NHS UK leaflets consistently achieved the highest scores across all conditions, indicating the easiest readability. Among LLMs, ChatGPT produced the most accessible text, while Gemini exhibited notably lower FRES scores, suggesting greater complexity and reduced readability.

**Figure 2:**
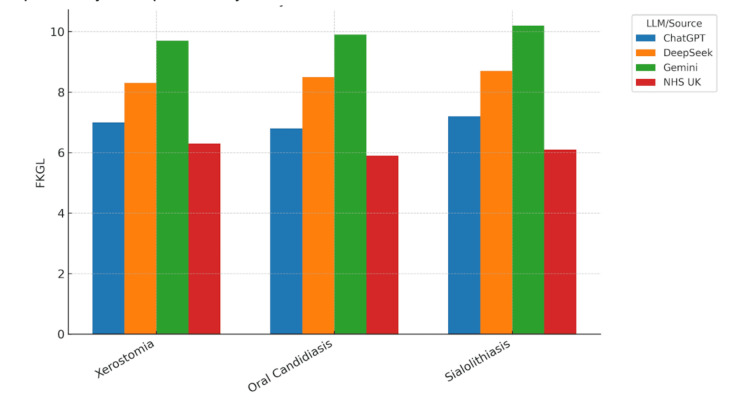
Flesch-Kincaid Grade Level (FKGL) scores for texts on xerostomia, oral candidiasis, and sialolithiasis generated by large language models (LLMs) and the NHS UK patient leaflets. Bar chart comparing Flesch-Kincaid Grade Level (FKGL) scores across three oral medicine conditions—xerostomia, oral candidiasis, and sialolithiasis—for texts generated by ChatGPT, DeepSeek, and Gemini, alongside the NHS UK patient information leaflets. Higher FKGL values indicate greater reading complexity and a higher educational level required for comprehension. NHS UK leaflets consistently demonstrated the lowest FKGL scores, suggesting they are substantially easier to read than text generated by large language models, particularly Gemini.

Figure 2 shows the FKGL scores, revealing significant variation in reading complexity across sources. The NHS UK leaflets consistently displayed the lowest FKGL values, between 5.9 and 6.3, corresponding to UK Year 6 to 7 reading levels. ChatGPT outputs were moderately higher, typically between 6.8 and 7.2, while DeepSeek and Gemini frequently exceeded grade level 8, with Gemini reaching values up to 10.2. These results indicate that NHS UK materials are substantially easier to read than AI-generated texts, particularly those produced by Gemini.

**Figure 3:**
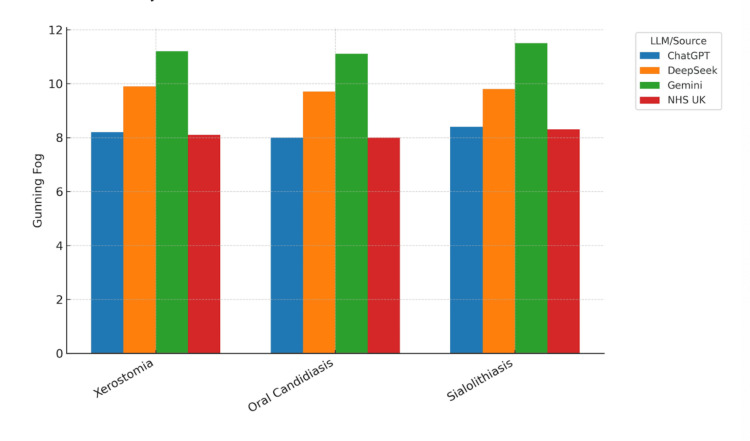
Comparison of the Gunning Fog Readability Index scores for patient information texts across three oral medicine conditions and multiple sources. Bar chart comparing the Gunning Fog Readability Index scores across three oral medicine conditions—xerostomia, oral candidiasis, and sialolithiasis—for texts generated by ChatGPT, DeepSeek, and Gemini, alongside the NHS UK patient information leaflets. Higher Gunning Fog scores indicate greater reading complexity due to longer sentences and increased use of polysyllabic words.

Figure 3 shows the Gunning Fog Index scores, which reflect sentence length and polysyllabic word use, were lowest for NHS UK materials, ranging from 8.0 to 8.3 across conditions. This suggests language suitable for readers around the UK Year 9. ChatGPT outputs were slightly higher, between 8.0 and 8.4, while DeepSeek and Gemini recorded significantly higher scores, often exceeding 9.5 and reaching up to 11.5 for Gemini. These findings confirm that Gemini produced the most linguistically complex and potentially less accessible patient information across all readability metrics.

## Discussion

Across all metrics assessed, the NHS UK patient leaflets consistently demonstrated superior readability compared to text generated by LLMs. These results held true for all three oral conditions examined: xerostomia, oral candidiasis, and sialolithiasis. Among the LLMs, ChatGPT produced the most readable outputs, with FKGL scores typically ranging from 6.8 to 7.2. Gemini generated the most complex materials, often exceeding an FKGL of 9.9 and reaching a Gunning Fog Index as high as 11.5 for sialolithiasis.

These findings are significant given that the National Institute for Health and Care Research (NIHR) recommends that patient-facing health information is written at or below Key Stage 3 reading levels, corresponding to a reading age of approximately 11-14 years [24,25]. Our analysis suggests that, while ChatGPT generally produced materials approaching this threshold, DeepSeek and especially Gemini frequently generated content requiring higher literacy skills. This discrepancy raises important questions about the suitability of AI-generated materials for patients with lower health literacy.

Health literacy remains a critical determinant of health outcomes. Approximately 43% of working-age adults in England experience difficulties understanding written health information unless it is presented in simple language; this figure rises to 61% when numerical data is included [26]. For individuals managing chronic conditions such as xerostomia, oral candidiasis, or sialolithiasis, clear and accessible written materials are essential to enable self-care, recognise complications, and know when to seek professional help. The risk is that patient materials exceeding recommended readability levels may inadvertently widen health inequalities [27,28].

Our findings also raise considerations regarding the potential for AI to contribute to digital health inequalities. While LLMs offer the promise of scalable, personalised patient information, there remains a risk that reliance on AI-generated materials could exacerbate disparities if the outputs are not adequately tailored for diverse literacy levels. The NHS Long Term Plan envisions digital health solutions that are inclusive and equitable [2]. Achieving this goal will require robust quality control mechanisms to ensure AI-generated content meets established readability standards and remains clinically accurate.

Several limitations must be acknowledged. Our analysis was based on single outputs from each LLM per condition, reflecting the finite number of free prompts obtainable during a single session; future studies should explore variability across multiple prompts and model iterations. Readability metrics, while useful proxies for linguistic complexity, cannot capture other important aspects of health communication such as cultural appropriateness, engagement, and trust. We also focused deliberately on first-pass outputs generated from a single neutral prompt to allow fair comparison between models and to reflect a plausible scenario in which users take the initial response as presented. While some individuals may choose to re-prompt for clarification, this behaviour is not universal and may depend on factors such as confidence in interacting with AI, comfort with the subject matter, or awareness that simplification is possible. Assessing first-pass outputs, therefore, provides a consistent and realistic benchmark for the readability of AI-generated health information.

Despite these limitations, our findings have important implications for the integration of AI into patient education. While LLMs, particularly ChatGPT, demonstrate potential to produce moderately readable patient information, current outputs often remain less accessible than professionally authored NHS materials. As AI continues to evolve, collaborative efforts between technologists, clinicians, and health communication experts will be essential to ensure that digital health tools are not only technologically sophisticated but also equitable and understandable to all patients.

## Conclusions

Our findings demonstrate that while LLMs such as ChatGPT, DeepSeek, and Gemini can rapidly generate patient information leaflets on oral medicine topics, their readability remains consistently lower than professionally authored NHS UK materials. This gap is not merely academic; it has real consequences for patient understanding and health equity, particularly given NHS ambitions to integrate AI into healthcare communication.

LLMs hold considerable promise for scaling health information delivery, but without rigorous oversight and further development focused on readability and patient-centred design, they risk producing content too complex for many readers. As digital health tools become increasingly embedded in care pathways, ensuring that AI-generated materials are accessible to all patient groups must remain a priority.
